# Pharmacokinetics of monoclonal antibodies and Fc-fusion proteins

**DOI:** 10.1007/s13238-017-0408-4

**Published:** 2017-04-19

**Authors:** Liming Liu

**Affiliations:** Department of Pharmacokinetics, Pharmacodynamics and Drug Metabolism, MRL, West Point, PA 19486 USA

**Keywords:** monoclonal antibody (mAb), Fc-fusion protein, pharmacokinetics, FcRn, target-mediated drug disposition (TMDD), glycosylation, anti-drug antibody (ADA), human PK prediction

## Abstract

There are many factors that can influence the pharmacokinetics (PK) of a mAb or Fc-fusion molecule with the primary determinant being FcRn-mediated recycling. Through Fab or Fc engineering, IgG-FcRn interaction can be used to generate a variety of therapeutic antibodies with significantly enhanced half-life or ability to remove unwanted antigen from circulation. Glycosylation of a mAb or Fc-fusion protein can have a significant impact on the PK of these molecules. mAb charge can be important and variants with pI values of 1–2 unit difference are likely to impact PK with lower pI values being favorable for a longer half-life. Most mAbs display target mediated drug disposition (TMDD), which can have significant consequences on the study designs of preclinical and clinical studies. The PK of mAb can also be influenced by anti-drug antibody (ADA) response and off-target binding, which require careful consideration during the discovery stage. mAbs are primarily absorbed through the lymphatics via convection and can be conveniently administered by the subcutaneous (sc) route in large doses/volumes with co-formulation of hyaluronidase. The human PK of a mAb can be reasonably estimated using cynomolgus monkey data and allometric scaling methods.

## **INTRODUCTION**

The ground-breaking discovery of monoclonal antibody (mAb) technology by Kohler and Milstein in 1975 provided the possibility of creating antibodies as a class of therapeutics (Kohler & Milstein, 1975). The original obstacles of immunogenicity of murine mAbs in humans was overcome by the revolution in molecular biology in the 1980s, which enabled humanization of murine antibodies and, eventually, the successful development of fully humanized antibodies. Humanization greatly reduces a therapeutic antibody’s immunogenicity in humans, making chronic administration possible. Such advances in antibody technologies have resulted in an explosion in the development of therapeutic mAbs over the last decade. Today, more than 47 mAbs and derivative drugs have been approved for human use with many of them attaining blockbuster status (Ecker et al., 2015). Among the top ten selling drugs in 2014, five were monoclonal antibodies and one was an Fc fusion molecule, with each having an annual revenue over 6.5 billion US dollars (Nisen, 2015). It is estimated that in the near future, about 30% of the new drugs will be antibodies or antibody derivatives (Elvin et al., 2013). Antibody derivatives include Fc-fusion proteins, antibody-drug conjugates (ADCs), immunocytokines (antibody-cytokine fusions), and antibody-enzyme fusions.

The efficacy of a mAb is largely dependent on its pharmacological and pharmacokinetic properties. The pharmacological effects or pharmacodynamics (PD) of a mAb are best described as “What a drug does to the body”; a mAb binds to a target (e.g., receptors, soluble antigens, etc.) and induces either antagonistic (i.e., blocking or neutralizing) or agonistic effects (i.e., activating), triggering down-stream pharmacological effects, leading to efficacy and/or unwanted side effects. The pharmacokinetics (PK) of a mAb is best described by “What the body does to the drug”. The fate of a mAb *in vivo* will be determined by how the body handles it under physiological or pathological conditions. The clearance or half-life of a mAb will determine the body’s “exposure” to the mAb, which in turn will determine the extent of PD effects. The exposure-response (PK-PD) relationship determines the outcome of a drug’s effects on the body. Understanding this relationship is an essential and integral part of drug discovery and development.

Being large proteins of 150 kDa, mAbs possess some unique PK properties, making the discovery and development pathway of mAbs substantially different from that of small molecule drugs. The unique PK properties are determined by many factors related to an antibody’s structure and functions including FcRn mediated recycling, glycosylation patterns, overall charge and pI, target-mediated clearance, anti-drug antibody response, and off-target binding.

In this review article, general PK properties and the factors influencing the PK of mAbs and Fc fusion proteins will be discussed, in addition to PK topics related to preclinical and early clinical development of mAb drugs. The impacts of glycosylation on the PK and PD of mAbs and Fc-fusion proteins have been reviewed extensively elsewhere (Liu, 2015) and will be only briefly discussed here.

## **FACTORS INFLUENCING THE PK OF MAB AND FC-FUSION PROTEINS**

**Figure 1:**
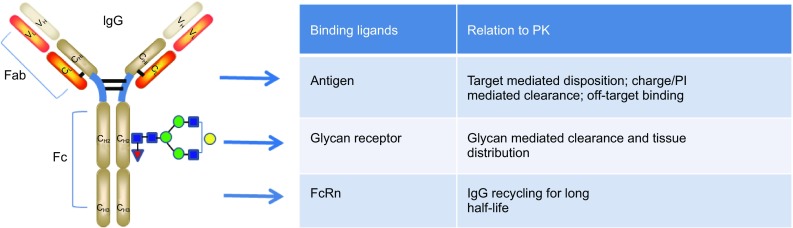
Antibody features that contribute to PK properties

An antibody is a complex molecule consisting of an antigen binding region (Fab domain) and a constant region (Fc domain). Fab binds to antigens and can be responsible for target-mediated clearance of a mAb. Glycosylation and charges in the Fab domain are also important for the PK properties of a mAb. In the Fc region, a subdomain of the C_H2_-C_H3_ domain is responsible for FcRn binding that results in recycling of antibodies for a long half-life (Roopenian & Akilesh, 2007). The same C_H2_-C_H3_ domain is also responsible for Protein A and G binding, which is exploited for antibody purification (Kim et al., 1994b). The C_H1_-C_H2_ domain is responsible for FcγR binding, which is critical for antibody effector functions (Daëron, 2014; Jefferis & Lund, 2002). A canonical glycosylation site is located in the Fc region C_H2_ domain (Asn297) in all IgG subclasses. Glycosylation patterns can impact both PK and PD significantly (Arnold et al., 2007; Jefferis, 2009a). The PK properties related to the structure of Fc are also relevant to Fc-fusion molecules. In addition, the fusion partner replaces Fab and may be responsible for target mediated clearance. Figure 1 depicts the general structure of IgG1 and the specific domains that are important for PK properties.

### **FcRn: key regulator of IgG PK**

**Figure 2:**
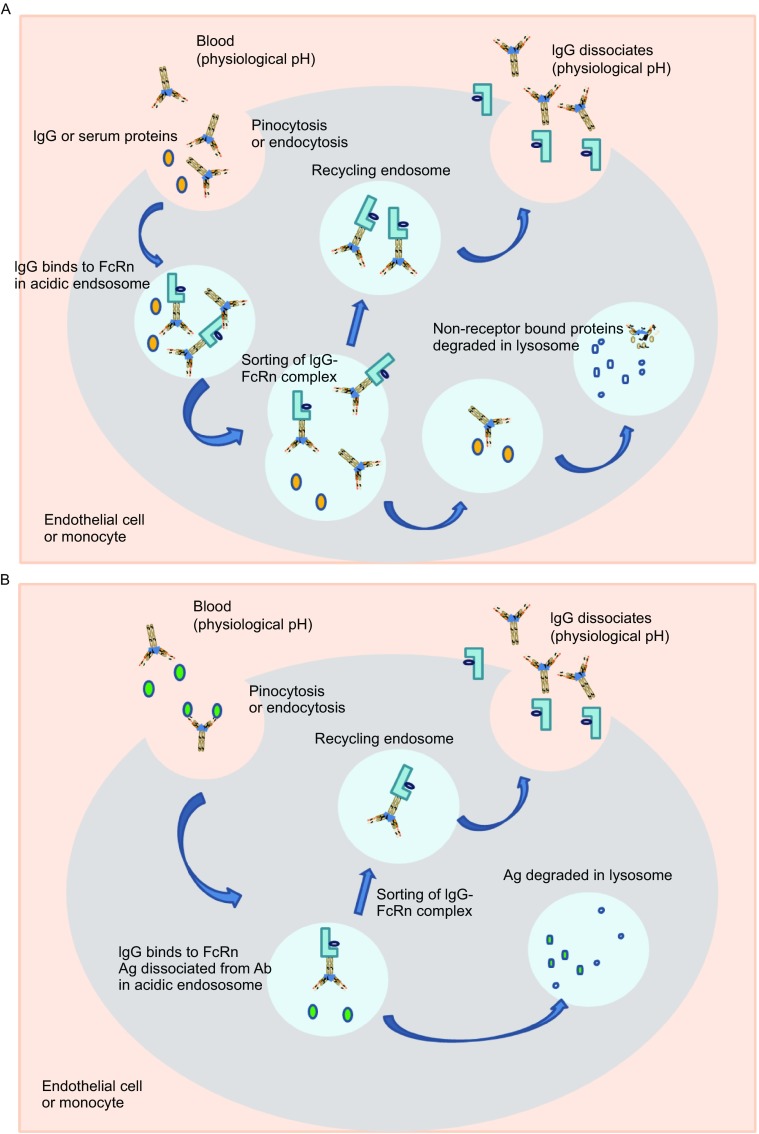
FcRn mediated IgG recycling pathway and antibody mediated antigen removal via pH dependent binding. (A) IgG circulating in the blood is taken up by endothelial cells or monocytes through either fluid phase pinocytosis or receptor mediated endocytosis. Once inside the cells, IgG binds to FcRn in the acidified endosomes. IgG that binds to FcRn migrates to the cell surface where the IgG encounters a physiological pH environment (~pH 7.4) and is released back into the blood. IgG that is not bound to FcRn (due to competition with other IgG) will be sorted to lysosomes for degradation. (B) mAb (with strong binding at pH 7.4 and no or weak binding at pH 6) binds to antigen at neutral pH in the circulation; once endocytosed into the cell and entered the acidic endosome, the antibody releases the antigen and binds to FcRn. FcRn bound antibody recycles back to the blood stream while the antigen is degraded in the lysosome

In the 1960s, Dr. Roger Brambell proposed a mechanism by which IgG is salvaged from catabolism by receptors located within cellular compartments and/or on the surface of cells (Brambell et al., 1964; Brambell, 1966). The “hypothesized” Brambell receptor, later called neonatal Fc receptor (FcRn), was cloned in 1989 (Simister & Mostov, 1989). Experiments in FcRn knockout mice have definitively confirmed that FcRn is responsible for protecting IgG from catabolism (Junghans, 1997; Roopenian & Akilesh, 2007). Over the past two decades, extensive work by many investigators has established the role of FcRn in regulating the levels and transport of IgG in the body (Roopenian & Akilesh, 2007), validating Brambell’s hypothesis. It is now established that FcRn binds to IgG at acidic pH (~6.0) with very low or negligible affinity at pH 7–7.4 (Ghetie & Ward, 1997; Roopenian & Akilesh, 2007), providing an ingenious biological solution to achieve exocytic release of recycled IgG. The binding area of IgG to FcRn is located between the C_H2_ and C_H3_ domain, distinctly different from the FcγR binding domain that is located between the C_H1_ and C_H2_ domain (Kim et al., 1994a; Roopenian & Akilesh, 2007). As depicted in Figure 2A, IgG circulating in the blood is taken up by endothelial cells or monocytes through either fluid phase pinocytosis or receptor-mediated endocytosis. Once inside the cells, IgG binds to FcRn in the acidic endosomes. IgG that binds to FcRn escapes lysosomal degradation and migrates to the cell surface where the IgG encounters a physiological (~pH 7.4) pH environment and is released back into the blood. IgG that is not bound to FcRn (due to competition of other IgG) will be sorted to lysosomes for degradation (Fig. 2A). Through competition of FcRn binding for recycling, the body can regulate IgG homeostasis: more IgG will be sorted to lysosomes for degradation when large amounts of IgG are present whereas more IgG will be rescued through recycling when the IgG concentration is low (Roopenian & Akilesh, 2007).

The elucidation of FcRn biology prompted active efforts by the biopharmaceutical industry to create therapeutic mAbs with super long half-lives through Fc engineering. The amino acids in the Fc region have been thoroughly investigated to identify mutations that significantly enhance binding to FcRn at pH 6 while maintaining little or no binding at neutral pH (7.4). It has been reported that specific Fc variants (T250Q/M428L, V308P, M428L, M252Y/S254T/T256E, M428L/N434S, N434A, N434H) that improve IgG affinity for FcRn at pH 6 with little or no binding to FcRn at neutral pH can lead to 2–4-fold longer terminal half-lives in cynomolgus and rhesus monkeys (Yeung et al., 2009, 2010; Vaccaro et al., 2005; Olafsen, 2012; Hinton et al., 2004,2006; Deng et al., 2010; Datta-Mannan et al., 2007a, b, 2012a, b). This general relationship has also been validated more recently in humans with the M252Y/S254T/T256E (YTE) set of mutations constructed on the Fc of a mAb against respiratory syncytial virus (RSV) with half-life increases from around 20 days to more than 60 days (Robbie et al., 2013). However, a good quantitative relationship between *in vitro* FcRn binding affinity and *in vivo* half-life has not been well-established (Datta-Mannan & Wroblewski, 2014), suggesting that factors other than FcRn interaction also play an important role. On the opposite end, reduction of IgG half-life can be realized for *in vivo* diagnostic and research purposes by engineering poor binders of FcRn (Swiercz et al., 2014; Olafsen et al., 2006). In addition, blockade of FcRn *in vivo* can decrease endogenous IgG half-life to treat autoimmune diseases (Patel et al., 2011; Liu et al., 2007; Challa et al., 2013).

FcRn binding affinity is one of the critical quality attributes (CQA) for therapeutic mAb manufacturing processes (Alt et al., 2016). Methionine in mAbs can be oxidized during manufacturing processes (Tsuchida et al., 2016). The Fc region of human IgG1 has three conserved methionine residues (Met252, Met358, and Met428), which are located near the C_H2_-C_H3_ interface where FcRn binds (Alt et al., 2016). It has been shown that the oxidation of these three Met residues impairs Fc–FcRn binding (Bertolotti-Ciarlet et al., 2009; Pan et al., 2009). When 79% of Met252 and 57% of Met428 were oxidized, the serum half-life of IgG decreased by about 83% in mice (Wang et al., 2011b). More recently, Gao et al. showed with mutagenesis analyses that either M252 or M428 oxidation alone significantly impaired Fc–FcRn binding. M252 oxidation generated a more deleterious effect on Fc–FcRn binding than M428 oxidation, whereas M358 oxidation did not affect Fc–FcRn binding (Gao et al., 2015).

Although it is the C_H2_-C_H3_ domain of Fc that is primarily involved in the binding to FcRn, the variable region (Fv) of Fab may also interact with FcRn and alter the interaction between IgG molecules and FcRn. Wang et al. found that although mAbs with identical Fc and different Fab exhibited a differential binding to FcRn at both pH 6 and pH 7.3, only the dissociation characteristics at pH 7.3 correlated with the *in vivo* PK properties of these mAbs (the slower the dissociation at pH 7.3, the shorter the half-life) (Wang et al., 2011a). More recently, Schoch et al. made similar observations with ustekinumab and briakinumab, both anti-IL12/IL23 mAbs, which have almost identical Fc but different Fab fragments (Schoch et al., 2015). The binding affinity to FcRn at pH 6 and the PI are very similar between these two mAbs, but there is a positive charge patch in the Fv of briakinumab, which renders strong binding to FcRn. The mAb therefore only dissociates from FcRn at higher pH, resulting in a shorter half-life for briakinumab (t_1/2_ ~9 days), in comparison to ustekinumab (t_1/2_ ~22 days) (Schoch et al., 2015). These results clearly indicate that the variable region does also influence IgG-FcRn interactions, thus impacting PK of a mAb.

Antibody and antigen complexes are also recycled through the FcRn pathway and can result in accumulation of bound antigens in the circulation and extension of the half-life of the antigens (Igawa et al., 2013). To effectively remove antigen from the circulation, it is desired that the bound antigens be degraded, while antibody is recycled. Through antibody engineering to render Fab binding to antigens pH-dependent (strong binding at pH 7.4 and no or weak binding at pH 6), Igawa et al. created an elegantly designed recycling antibody that directs antigens for lysosomal degradation while ensuring that the mAb is recycled to bind antigen again, thus reducing antigen concentrations (Fig. 2B) (Igawa et al., 2010a, 2013). Furthermore, they created a sweeping antibody through engineering pH-dependent antigen binding in the Fv region and higher binding affinity to FcRn or FcγRIIB in the Fc region. The sweeping antibody can bind to FcRn or FcγRIIb at neutral pH to facilitate the uptake of the immune complex; bound antigen (such as IL-6R) dissociates from the antibody for degradation once in the lysosome, resulting in a significantly reduced antigen concentration (Igawa et al., 2016). However, the sweeping antibodies are also cleared significantly faster than conventional antibodies, likely due to these antibodies’ ability to bind FcRn at neutral pH, which prevents them from being recycled back to the circulation (Igawa et al., 2016). It appears that the sweeping antibody targeting FcγRIIb for enhanced uptake does not have this drawback (Igawa et al., 2016).

Other factors related to FcRn functions that can impact the PK of a mAb include FcRn polymorphism and IgG allotypes. It has been shown that the polymorphism of the variable number of tandem repeats region in the FcRn promoter can influence the FcRn expression level and binding ability (Sachs et al., 2006). IgG allotype can also influence the binding affinity of a mAb to FcRn, with better binders having longer half-life, which may contribute to the variability of PK in the clinic (Ternant et al., 2016). For example, it has been shown that a mAb with the G1m17,1 allotype, such as infliximab, has better binding affinity for FcRn than those with the G1m3 with no G1m1 (G1m3,−1) allotype. In patients homozygous for the G1m17,1 allotype, infliximab is in competition with endogenous IgG with the G1m17,1 allotype. As a result, infliximab exhibits a shorter half-life in patients homozygous for the G1m17,1 allotypes than in those carrying the G1m3,−1 allotype (Ternant et al., 2016). In addition, baseline albumin level in the serum can be used to predict the functional status of FcRn *in vivo* in that the trough level of an administered mAb correlate positively with the baseline albumin concentration (Fasanmade et al., 2010).

Although FcγR binding is critical for IgG’s effector functions such as antibody dependent cell-mediated cytotoxicity (ADCC) and complement dependent cytotoxicity (CDC), FcγR does not appear to significantly affect IgG’s PK properties. This is likely due to the fact that a large pool of serum IgG can compete with the binding of relatively small amounts of mAb to FcγR, effectively ameliorating the impact of FcγR binding. Consistent with this, Abuqayyas and coworkers have shown that 8C2, a mouse IgG mAb, exhibited similar PK and tissue distribution in both FcγR knockout mice and in wild type mice (Abuqayyas et al., 2013).

### **Glycosylation impacts on PK of mAb and Fc-fusion proteins**

Like natural IgGs, all approved recombinant therapeutic mAbs are glycosylated, although some non-glycosylated mAbs or derivatives are in clinical development (Liu, 2015). Therapeutic mAbs or derivatives have an asparagine (Asn)-X-Ser/Thr (Where X is any amino acid except Pro) consensus sequence for *N*-glycosylation at the position Asn297 in the heavy chain of the C_H2_ constant domain. Some therapeutic mAbs also bear additional glycosylation in the Fab domain. For instance, cetuximab is glycosylated at Asn88 of the VH region (Jefferis, 2009b). In addition, some of the Fc-fusion partner molecules, such as etanercept and B cell activating factor receptor 3 (BR3)-Fc, also possess O-linked glycans (Pennica et al., 1993; Stefanich et al., 2008).

Characterization of aglycosylated IgG, produced through chemical modification or genetic engineering, confirmed that glycosylation is not required for an IgG antibody’s long half-life (Kim et al., 1994a; Liu et al., 2011; Nose & Wigzell, 1983; Tao & Morrison, 1989). The clinical evidence for aglycosylated IgG having normal PK is demonstrated by the mAb ALD518, a humanized anti-human IL-6 IgG1 produced in yeast. In a phase I clinical trial, the circulating half-life for ALD518 was 20–32 days, consistent with half-life of a normal human IgG1 (Clarke et al., 2009). Glycosylated mAbs with terminal high mannose glycans have been shown to exhibit fast clearance from the blood (Goetze et al., 2011; Kanda et al., 2007; Liu et al., 2011; Wright & Morrison, 1994; Wright et al., 2000; Yu et al., 2012). The fast clearance of mAbs containing high mannose has also been demonstrated in clinical studies (Chen et al., 2009; Goetze et al., 2011). Glycan receptors that have been attributed to the removal of glycoproteins *in vivo* include the mannose receptor (ManR) and the asialoglycoprotein receptor (ASGPR). The ASGPR and the ManR are carbohydrate-specific, endocytic receptors expressed by hepatic parenchymal (hepatocytes) and nonparenchymal (such as Kupffer) cells, and sinusoidal endothelial cells, respectively (Ashwell & Harford, 1982; Mi et al., 2014).

The shorter half-life of an Fc-fusion molecule in comparison to the whole IgG has been attributed to the lower binding affinity to FcRn, the glycan mediated disposition and the receptor (of fusion partner) mediated disposition (Liu, 2015). Among these attributes, glycosylation patterns may play a more important role in determining the *in vivo* clearance of Fc-fusion molecules. For example, in the investigation of humanized yeast-produced TNFαRII-Fc-fusion molecules, it was demonstrated that it was the extent of sialylation on the TNFRII, not the FcRn-binding affinity, which determined the clearance. The exposure was positively correlated to the quantity of the sialylation on the receptor molecule, with higher sialic acid content resulting in higher exposure (Liu et al., 2013).

Most of the Fc-fusion molecules including BR3-Fc, IL-23R-Fc, CTLA4-Ig, and LFA3TIP rely on sialylation in reducing the *in vivo* clearance (Liu, 2015). Other terminal monosaccharide, such as GlcNAc, can also contribute to the PK properties of Fc-fusion molecules (Keck et al., 2008; Jones et al., 2007).

### **Impact of charge and pI on mAb PK**

Isoelectric point (pI), a measurement of protein charge, is defined as the pH at which the protein carries no net electrical charge. In general, antibodies that are chemically or genetically modified to achieve higher, more basic, pI values exhibit a high propensity to adhere to anionic sites of cell surfaces, resulting in increased tissue uptake and fast clearance from circulation. In contrast, modified antibodies with lower, more acidic pI values have a lower rate of uptake into cells (as a result of repulsion from the negatively charged cell surface), leading to decreased tissue uptake and blood clearance (Boswell et al., 2010; Bumbaca et al., 2012, 2015; Igawa et al., 2010c; Kobayashi et al., 1999; Lee & Pardridge, 2003). Chemically modified Fabs with a pI reduction of 1–2 units showed decreased blood clearance and tissue accumulation relative to the unmodified Fab (Kobayashi et al., 1999). With antibody pI variants generated using site-directed mutagenesis in the Fab region, Igawa et al. demonstrated that variants with pI values of 1–2 units lower than the wild type displayed longer half-lives and reduced clearance following both subcutaneous and intravenous administration (Igawa et al., 2010b). The data demonstrated that the clearance was positively correlated with pI, i.e., the half-life was negatively correlated with pI. Similar data was obtained from both human and minipig model in that antibodies with higher pI values exhibited faster clearance and lower subcutaneous bioavailabilities than antibodies with lower pI values (Zheng et al., 2012). Manipulation of pI was also used to reduce the toxicity of immunotoxin. Onda et al. showed that reducing the pI of the Fv portion of an immunotoxin significantly reduced liver toxicity, presumably reducing the distribution to the liver (Onda et al., 1999).

Charge variations can arise from antibody manufacturing processes due to chemical or enzymatic degradation via oxidation, deamidation, isomerization, and fragmentation. The charge differences may impact both PK and PD. To investigate whether mAb charge variants possess different PK and PD properties, Khawli et al. isolated basic, neutral, and acidic variants from a mAb product and characterized them in PK and PD assays. Their results showed that all variants had similar potency in PD assays and rat FcRn binding relative to the starting material. Following iv or sc administration in rats, no difference in serum PK was observed, indicating that pI differences among charge variants were not sufficient to result in PK changes. However, it is worth noting that the pI difference between the acidic variant and the basic variants were less than 0.5 (8.7 vs. 9.1) (Khawli et al., 2010). Based on the above evidence, it can be concluded that shifts in isoelectric point of approximately one pI unit or more are likely to produce measurable changes in PK and tissue distribution; differences of less than one pI unit are mostly inconsequential to PK.

**Figure 3:**
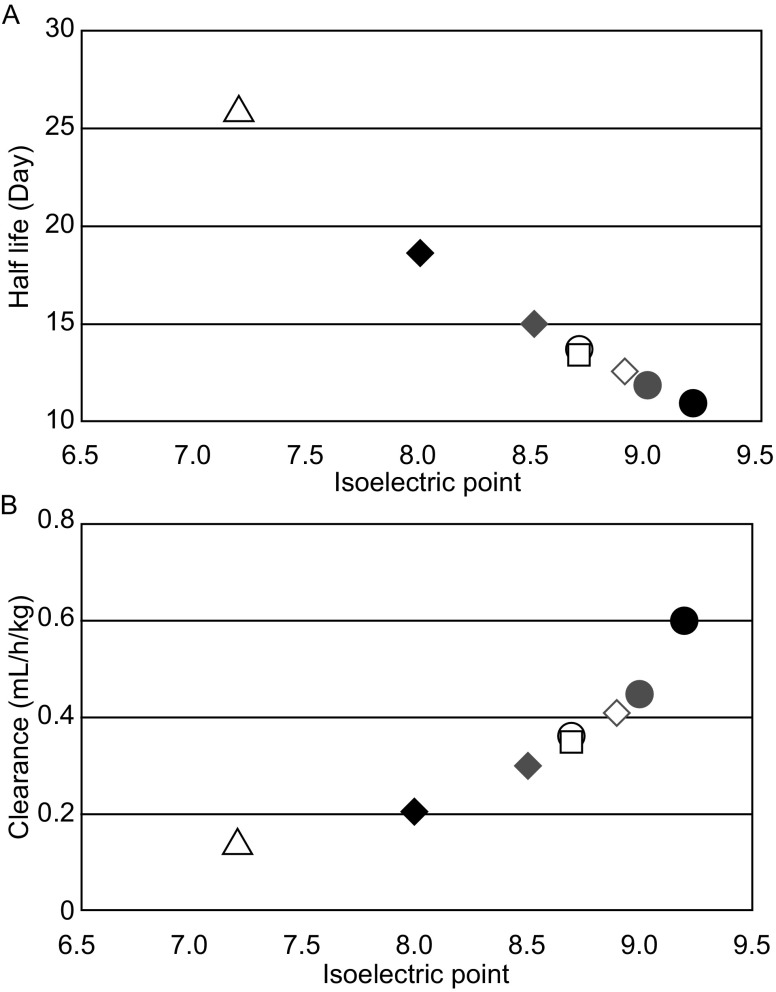
Relationship between charge/pI and half-life and clearance in PK of mAbs. Reproduced from Igawa et al., with permission (2010b)

Changing the pI of a mAb is a potentially powerful way to improve PK properties of some mAbs, especially those having strong target-mediated clearance, such as antibodies targeting surface receptors. For example, Igawa et al. modified the variable region to reduce the pI of an anti-IL6R mAb, resulting in a 2.4-fold half-life extension and 4.4-fold reduction in clearance (Igawa et al., 2010b). With mAb pI ranging from 7.2 to 9.2, they demonstrated a strong relationship between pI and PK (half-life and clearance) (Fig. 3). Sampei et al. identified a positive charge patch on an anti-FIXa antibody that was implicated in causing fast clearance. They introduced a negatively charged amino acid (Tyr30glu) near the patch and observed a significant improvement in PK (Sampei et al., 2013). More recently, Li et al. showed that an humanized anti-HCV mAb with human kI and VH3 framework exhibited unexpectedly fast clearance that was related to a higher framework pI rather than FcRn binding or target-mediated disposition (Li et al., 2014). A humanized mAb with framework modification resulting in lower pI had significantly improved PK profile (Li et al., 2014). They also confirmed that high pI mAb exhibited higher distribution to liver and spleen than did low pI mAb.

It is worth noting that the above observations on the impact of pI on PK were based on work with mAbs against the same targets. mAbs that bind to different targets may not be directly comparable based on pI alone; individual mAbs against different targets have their own physicochemical characteristics that may uniquely contribute to PK properties. For instance, Bevacizumab, a humanized mAb against VEGF, with a pI of 8.2 due to kI/VH3 scaffold, exhibited normal PK in rats whereas the aforementioned anti-HCV mAb, with pI of 8.2 with the same framework, exhibited poor PK (Li et al., 2014). In addition, for a panel of 13 antibodies humanized on a kI, VH3 scaffold, clearance in rats is not correlated with pI (Li et al., 2014). Furthermore, Hotzel et al., testing a larger panels of mAbs in cynomolgus monkeys, found that PK was not correlated with pI although the pI of the mAbs tested was narrowly clustered, between 7.5 and 9.5 (Hotzel et al., 2012a).

### **Impact of target-mediated drug disposition on mAb PK**

Target-mediated drug disposition (TMDD) is a nonlinear PK phenomenon, where drug–target binding (complexation with receptors, enzymes, or transporters) and subsequent events (dissociation and drug-target complex degradation) result in dose-dependent changes in PK (Mager & Jusko, 2001). In comparison to small molecule drugs, TMDD is more common for mAb drugs, especially for those targeting membrane receptors. It was estimated that half of the marketed mAbs exhibit TMDD (Dirks & Meibohm, 2010; Mould & Sweeney, 2007). The actual number is probably higher because the clinical doses at which drugs are studied often saturate the target mediated elimination pathway, thereby masking the nonlinear PK.

**Figure 4:**
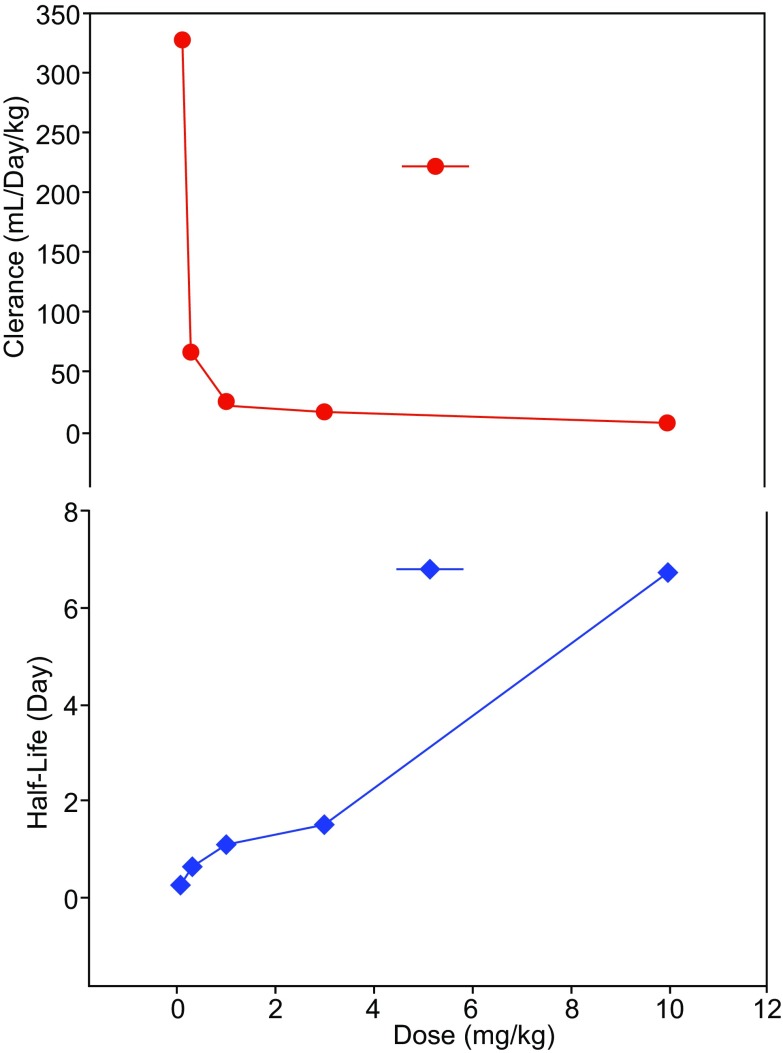
A typical TMDD graph exhibiting the relationship between clearance or t_**1/2**_ and mAb dose. Anti-α_v_ mAb was infused to cancer patients; clearance or terminal half-life (t_1/2_) was plotted against dose (0.1, 0.3, 1, 3, and 10 mg/kg). Adapted from Mullamitha et al., Clin Cancer Res. (2007)

At low concentrations TMDD accounts for a significant portion of mAb clearance. As mAb concentrations increase, target mediated elimination starts to saturate and clearance decreases dramatically. At high mAb concentrations, the clearance approaches a first-order process where the FcRn mediated pathway is dominant and the nonlinear pathway becomes negligible. To determine whether a mAb undergoes TMDD, a single ascending dose iv PK study in a pharmacologically relevant species (in which the mAb binds to the target of interest) is recommended. A typical characteristic of TMDD is readily recognizable by plotting clearance or t_1/2_ against dose as depicted in Fig. 4 (Mullamitha et al., 2007).

Nonlinear PK caused by TMDD may have significant consequences on study design (both pre-clinical and clinical), in particular on dose selection, dosing scheme, and sampling times (Dirks & Meibohm, 2010; Grimm, 2009). Preclinical PK studies with mAb for which TMDD is suspected must be conducted in pharmacologically relevant species (often in NHP) to determine whether the drug is subjected to TMDD. However, the interpretation of the results may be biased by anti-drug antibodies (ADA) interference. It is preferable to conduct a PK study with PD measurement to estimate the exposure and response relationship and to determine whether the PK saturating dose is also the dose at which the PD is saturated. However, it is important to bear in mind that preclinical data can only give a rough estimate of the saturating dose of a mAb and that findings may not be directly translatable to the clinic. It is generally appreciated that preclinical information is a starting point for designing a clinical TMDD study.

Although TMDD generally occurs more frequently for mAbs that target surface antigens, mAbs against soluble antigens can also exhibit TMDD. For example, Brenner et al. showed that FG-3019, a human mAb against connective tissue growth factor (CTGF) exhibited fast clearance that was determined to be target related. The fast clearance of the FG-3019-CTGF complex was due to the extremely fast clearance of CTGF itself, which is cleared mainly through liver uptake (Brenner et al., 2016).

Because of its assumption of linear disposition, the non-compartmental analysis (NCA) method is inappropriate for PK analysis of drugs with TMDD, except perhaps for initial exploration to uncover or confirm TMDD. While steady state of volume distribution (Vss) can be inferred from blood concentration using NCA for small molecules, Vss for mAb may be underestimated and incorrect if it is derived from NCA analysis or simple compartmental analysis. This is because these models assume linear PK and rapid equilibrium between blood and tissues (Wang et al., 2008). Therefore, more sophisticated mechanistic models are needed for the TMDD analysis. A general framework of a mechanistic TMDD model was established by Mager and Jusko (2001). Following the base model introduction, several similar models have been developed in several variations (Dua et al., 2015; Gibiansky et al., 2008; Grimm, 2009; Mager, 2006). Information on the target pharmacology, in particular its expression and turnover, drug-target binding kinetics, and knowledge of the fate of drug-target complexes, are generally required to build a mechanistic model. However, in most situations, this information is not readily available. For this reason, many analyses use empirical approaches for building models that require less information about the drug target interaction, receptor numbers, and turnover rates (Dua et al., 2015; Gibiansky et al., 2008; Grimm, 2009; Mager, 2006). Nevertheless, in most situations, at least one of the variant models can be useful in modeling a specific mAb’s nonlinear behavior.

Based on the model of quasi-steady-state approximations, Grimm thoroughly analyzed the consequences of TMDD on the disposition of mAb (Grimm, 2009). Several conclusions were drawn from this analysis. First, saturation of clearance (from PK analysis) signifies the saturation of the targets when the target is readily accessible. A mAb targeting soluble antigens in the circulation is one example of this situation. Second, when the target is only slowly accessible due to low permeability, saturation of clearance may not imply the saturation of targets, although it may still be used as a proxy for the saturation of the target. Third, when the site of target-mediated clearance and the desired site of action are not the same, especially when the latter is poorly accessible, saturation of clearance no longer implies on-site target saturation. This may occur, for example, when targeting a receptor on a solid tumor which is poorly accessible, and the receptor is also expressed on other normal tissues (such as vascular endothelium) (Grimm, 2009). It has been observed in many cases that *in vitro* *K*_d_ of a mAb is often different from the *in vivo* *K*_d_ (or *K*_m_) obtained from modeling (Brenner et al., 2016; Ng et al., 2006). The *in vitro* *K*_d_ often overestimates the affinity of the mAb, likely due to the simplified assay system. The complex situation *in vivo* can impact the apparent *K*_d_ differently (Brenner et al., 2016; Ng et al., 2006). Other factors such as fast *K*_int_ (drug-target internalization rate constant), fast target turnover and limitation of accessibility or permeability also play important roles in drug and target interactions (Grimm, 2009). When choosing clinical candidates, what is the optimum affinity for a mAb? Based on Grimm’s analysis, in most cases, optimization of an antibody is a matter of optimizing *K*_m_. In certain situations (e.g., *K*_off_ ≪ *K*_int_), increasing affinity by decreasing *K*_off_ may yield no benefit because of the fast internalization of the target (Grimm, 2009).

### **Impacts of anti-drug antibody (ADA) on mAb PK**

Most, if not all, therapeutic proteins including mAbs, are immunogenic and can induce ADA in humans. The incidence and the impacts on PK/PD and efficacy vary from drug to drug. Immunogenicity of therapeutic mAbs can cause hypersensitivity responses such as anaphylaxis and infusion reactions, and accelerated clearance of drug, resulting in decreased safety and efficacy (Chirmule et al., 2012).

Both humanized and fully human mAbs can induce ADA in humans; there is no evidence that fully human antibodies are less immunogenic than humanized ones. For example, Humira^®^ (adalimumab), a fully human mAb, induces ADA and impacts PK and efficacy in patients (Bartelds et al., 2011; Chirmule et al., 2012; Harding et al., 2010). Bartelds et al. reported that ADA was developed in 28% of 272 rheumatoid arthritis (RA) patients who were treated with Humira^®^ for 3 years. RA patients without anti-adalimumab antibodies had much higher adalimumab concentrations (median, 12 mg/L) compared with patients with antibody titers from 13 to 100 AU/mL (median, 5 mg/L) (Bartelds et al., 2011). There were significantly more treatment failures that led to the discontinuation of the treatment in patients who developed ADA (Bartelds et al., 2011).

**Figure 5:**
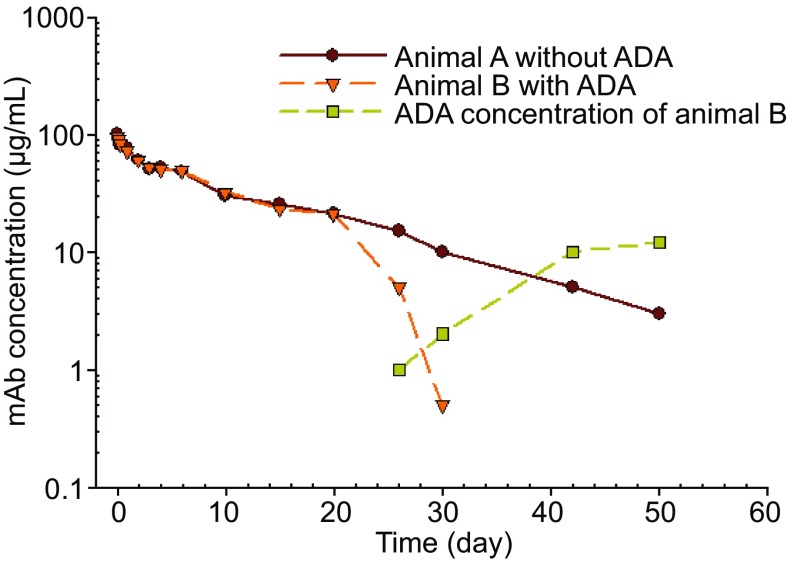
Hypothetical concentration time curves following iv administration of a mAb in animals with or without ADA

Human mAb or humanized mAb are immunogenic in animals. Van Meer et al. examined the immunogenicity of 27 marketed mAb drugs in NHP and 25 (93%) developed ADA to the administered mAb (van Meer et al., 2013). ADA can cause perturbation of pharmacokinetic profiles, making it difficult to interpret PK/TK data, particularly when TMDD is suspected. The simplest way to determine the cause of abnormal PK when both ADA and TMDD are the suspects is to measure ADA levels in the serum or plasma of the dosed animals. In most cases, clearance likely coincides with the presence of ADA (Richter et al., 1999). However, the absence of measurable ADA does not mean the absence of ADA especially when the ADA assay sensitivity is low and/or when the drug concentration is above the assay’s drug tolerance level (Kelley et al., 2013). Figure 5 shows the typical impact of ADA on the clearance of an administered mAb drug.

In most cases, ADA occurs mainly in animals exposed to lower doses rather than higher doses. The reason for this phenomenon may be related to high dose induced tolerance (high zone tolerance) (Gilliland et al., 1999; Somerfield et al., 2010) or the interference of high concentrations of mAb in the serum. Since ADA can lower the exposure of the drug in toxicity species, it is important to use sufficiently high dose levels to achieve desired exposure multiples (based on the anticipated clinical dose). It is also important to take into consideration the ADA assay performance in terms of drug tolerability before declaring a lack of ADA.

The occurrence of ADA of human mAb can be rapid or slow in preclinical animal species. Typically, ADA may appear 2–3 weeks after single dose administration and these are mostly IgG response. In some cases, the occurrence can be even earlier which most likely is mediated by IgM subclass. It is important to note that immunogenicity or ADA response found in preclinical species does not predict ADA response in human (van Meer et al., 2013). It has been reported that the incidence of ADA formation in NHPs and patients was comparable in only 59% of the cases (van Meer et al., 2013). mAbs can be very immunogenic in animals and yet show no apparent or negligible immunogenicity in humans. Keytruda (pembrolizumab), a humanized IgG4 anti-PD1 mAb for the treatment of multiple cancers, is a good example of species differences in ADA formation. Keytruda is quite immunogenic in cynomolgus monkeys as a high incidence of ADA was observed. (BLA# 125514Orig1s000 Keytruda FDA review package, pharmacology. August 22, 2014; Link http://www.accessdata.fda.gov/drugsatfda_docs/nda/2014/125514Orig1s000PharmR.pdf).

However, in clinical studies in patients treated with Keytruda (2 mg/kg every 3 weeks, 200 mg every 3 weeks, or 10 mg/kg every 2 or 3 weeks), only 20 (1.7%) of 1,149 evaluable patients tested positive for treatment-emergent anti-pembrolizumab antibodies. There was no evidence of an altered pharmacokinetic profile or increased infusion reactions with anti-pembrolizumab binding antibody development (Highlights of Prescribing Information of Keytruda; Revised: 08/2016. https://www.merck.com/product/usa/pi_circulars/k/keytruda/keytruda_pi.pdf).

### **Impacts of off-target binding on mAb PK**

Off-target binding can significantly affect the PK, tissue distribution, efficacy, and toxicity of therapeutic antibodies. Although antibodies developed against an antigen are highly specific to that antigen, some mAbs can have multi-specificity binding capability for irrelevant antigens (James et al., 2003; Notkins, 2004). To prevent autoimmune diseases, antibodies matured through somatic mutations go through stringent control processes *in vivo* to weed out those unfavorable mutants that bind non-specifically to self-antigen or interact broadly with normal tissues. However, antibodies derived from *in vitro* selection or maturation lack an *in vivo*-like control system and rely on the counter screen to ensure specificity. This can lead to the selection of some non-specifically binding variants. For example, the A4B4 variant, (a prototype of Motavizumab, which is an *in vitro* affinity matured anti-RSV mAb derived from palivizumab), demonstrated broad tissue binding and accelerated clearance *in vivo* (Wu et al., 2007). Multiple off-target interactions were demonstrated on protein biochips for adalimumab, which was derived *in vitro* from a cloned human antibody phage library (Feyen et al., 2008).

Recently, Datta-Mannan et al. showed that bispecific IgG-scFv or IgG-ECD antibodies were cleared rapidly through liver sinusoidal endothelial cells (LSEC) in the liver (Datta-Mannan et al., 2016). The rapid clearance of BsAb was not attributed to TMDD, reduced Fc-FcRn interaction, or poor molecular or biochemical properties. Instead it was due to non-specific binding or association with LSEC. mAbs may also bind specifically to a non-intended target and cause alteration of PK/PD and toxicity profiles. Bumbaca et al. reported that a humanized mAb (hLD1.vB) directed against fibroblast growth factor receptor (FGFR) 4 exhibited specific binding to mouse complement component 3 (C3), leading to accelerated clearance and limited efficacy in mice (Bumbaca et al., 2011). Vugmeyster et al. also reported that an anti-monkey A-beta mAb had off-target binding in cynomolgus monkeys, leading to accelerated clearance (Vugmeyster et al., 2011).

Because the unpredictability of the off-target binding, it is recommended that mAb candidates go through early screens for non-target bindings *in vitro* including protein microarrays, light directed peptide synthesis arrays, and tissue cross-reactivity assays (Kelly et al., 2015; Hotzel et al., 2012b). These screens, along with preclinical evaluation in rodents and primates, are important tools in the assessment of how well a mAb will behave in the clinic. Hotzel et al. reported a strategy for mitigation of risk for antibodies with fast clearance. It is an assay based on ELISA detection of non-specific binding to baculovirus particles that can identify antibodies having increased risk for fast clearance (Hotzel et al., 2012b). This assay can be used during lead generation or optimization to identify antibodies with increased risk of having fast clearance in both humans and cynomolgus monkeys, thus increasing the likelihood of obtaining a suitable drug candidate. Recently high throughput cross-interaction assays were developed to screen mAbs for off-target mediated clearance. These assays showed high correlation with clearance rate in mice (Kelly et al., 2015). Others used protein and/or tissue arrays to determine cross reactivity of mAbs (Lueking et al., 2008; Kijanka et al., 2009).

## **MAB ABSORPTION**

The majority of FDA-approved therapeutic antibodies are administered intravenously (iv). The iv route allows for rapid delivery of large amounts of antibodies to the systemic circulation with complete systemic availability. In addition, the iv route allows the administration of larger volumes in comparison to other parenteral routes of administration. However, iv delivery, often required to be conducted in hospitals or doctor’s office, is not convenient for patients, and increases the cost of therapy. In addition, rapid infusion of antibody may also induce adverse events such as infusion reactions. Therefore, for some mAbs requiring chronic dosing, extravascular routes such as subcutaneous (sc) and intra-muscular (im) administration have been developed. Sc or im administration can be performed by a health care professional in a patient’s home or even by self-administration (Richter & Jacobsen, 2014). Examples for extravascular administration of mAbs or Fc fusions are adalimumab (sc), alefacept (im), efalizumab (sc), etanercept (sc), omalizumab (sc), and palivizumab (im).

The systemic absorption of antibodies following sc or im administration most likely occurs via the lymphatic system. The highly porous lymphatic system allows the transport of macromolecules (>20 kDa MW) through the convective flow of the interstitial fluids (Richter et al., 2012; Richter & Jacobsen, 2014). Since the lymph fluid drains slowly into the vascular system, the absorption of antibodies from the site of administration usually continues for hours or even days (Richter et al., 2012). In animals and in man, the time to maximal plasma concentrations (T_max_) of antibody are typically observed 1–8 days following sc or im administration (Lobo et al., 2004). In general, antibodies given by im or sc are well absorbed, resulting in bioavailabilities ranging from 50% to 100% (Richter & Jacobsen, 2014). There is no correlation between MW and the bioavailability of protein therapeutics in several animal species and in humans (Richter et al., 2012).

The extent of absorption of mAbs can be variable depending on the extent of pre-systemic antibody degradation including proteolytic degradation, endocytosis at the injection site and rates of recycling through interaction with FcRn (Wang et al., 2008). The absorption of therapeutic proteins can also be impacted by formulation and injection volumes (Richter et al., 2012; Richter & Jacobsen, 2014). In addition, physiologic factors such as age, body weight, movement, heat, and injection site may also serve as covariates for the bioavailability of therapeutic proteins including mAbs (Richter & Jacobsen, 2014). It was observed that fat content and physical activities can profoundly impact the absorption of therapeutic proteins in an animal model (Wang et al., 2012). Sc absorption rates in animals appear to be more rapid than in humans. Non-human primates tend to overestimate the sc bioavailability of mAbs and rodent and several other preclinical species do not appear to exhibit any clear patterns in relationship to humans (Richter et al., 2012). Recently the minipig was suggested as a potential translatable model for mAb pharmacokinetics following iv or sc administration (Zheng et al., 2012).

Large doses may not be feasible for sc or im administration due to the relatively limited solubility of IgG (~100 mg/mL); small volumes can be administered via these routes (2.5 or 5 mL for sc and im per site, respectively) (Richter et al., 2012). For mAbs requiring relatively low dose, such as Humira (40 mg/dose), sc administration can be easily achieved at 40 mg/0.8 mL injection pen (Humira^®^ PI). However, for mAbs requiring high doses, such as omalizumab, multiple injection sites are necessary; a dose of 375 mg is delivered via three separate 1 mL sc injections (Xolair PI). More recently, the limitation of volume in sc administration of mAb appears to have been ameliorated by including hyaluronidase in the formulation. In the subcutaneous space, the glycosaminoglycan hyaluronan, together with collagen, creates a volume barrier within the extracellular matrix (Bookbinder et al., 2006). Hyaluronan can be degraded by hyaluronidase and hyaluronidase derived from animal testes has been used extensively to facilitate dispersion of co-injected materials in the clinic (Bookbinder et al., 2006). With co-formulation of hyaluronidase, it is now clinically feasible to administer mAb’s for oncology indications which typically require much larger doses and volumes (Jackisch et al., 2014; Salar et al., 2014; Shpilberg & Jackisch, 2013). rHuPH20, a recombinant hyaluronidase temporarily cleaving hyaluronan fibers in the interstitial tissue, and thereby facilitating the sc injection of large volumes, has been used to co-formulate several important anti-cancer mAbs. With co-formulation with hyaluronidae, it is now possible to administer volumes up to 5 mL sc in approximately 5 min for 600 mg trastuzumab (Bittner et al., 2012). Rituximab can be delivered in 2–8 min with 4.4–15.0 mL sc (Salar et al., 2014). A fixed dose of 1,400 mg of rituximab was successfully delivered via sc injection by hyperconcentrating rituximab (12-fold) relative to the IV formulation and including the rHuPH20 as an excipient in a volume of 11.7 mL (Salar et al., 2014; Shpilberg & Jackisch, 2013). The C_trough_ of sc delivered mAb was not inferior to that delivered by IV at 375 mg/m^2^ (Salar et al., 2014). Sc administration is also generally more immunogenic than drug dosed via the IV route. For example, anti-trastuzumab antibodies were detected in 8 of the 58 patients who received trastuzumab sc compared to zero of the 12 patients who received trastuzumab IV. That said, the presence of anti-trastuzumab antibodies did not correlate with the adverse events and pharmacokinetic profiles in these patients (Wynne et al., 2013). In EU, sc formulation for rituximab was approved for NHL (1,400 mg) in 2014 and for CLL (1,600 mg) in 2016 (Roche Media Release, 2016). Herceptin sc formulation (600 mg) for breast cancer was approved in EU in 2013 (Roche Media Release, 2013).

## **MAB DISTRIBUTION**

Being large and polar molecules, antibodies move very slowly across vascular endothelial cells. Convection is believed to be the primary mechanism responsible for the transport of antibody from blood to the interstitial space of tissues (Richter & Jacobsen, 2014). The rate of convection out of the blood and the rate of antibody catabolism within tissue determine the rate of antibody elimination. In general, antibody concentrations in tissue interstitial fluid are substantially lower than antibody concentrations in plasma because of the differences in the rate of convective uptake (which is slow) and elimination of antibody from tissue (which could be fast). However, higher antibody concentrations have been observed in tissues that are highly perfused and with relatively leaky vasculature (e.g., bone marrow, spleen, and liver). IgG antibodies show very little distribution to the brain because of blood brain barrier. Shah and Betts conducted analyses of bio-distribution data of non-binding mAbs from several different species (mice, rat, monkey, and human). Using a physiologically based pharmacokinetic (PBPK) model, they were able to define the relationship between plasma and various tissue concentrations as the antibody biodistribution coefficient (ABC). They found that typically the concentration of mAb in lung is 15%, heart 10%, kidney 14%, muscle 4%, skin 16%, small intestine 5%, large intestine 5%, spleen 13%, liver 12%, bone 7%, stomach 5%, lymph node 8%, adipose 5%, brain 0.4%, pancreas 6%, testes 6%, thyroid 68%, and thymus 7% of the plasma concentration (Shah & Betts, 2013). These results are consistent with the general estimates of tissue to blood ratio range of 0.1 to 0.5 for mAbs (Lobo et al., 2004). However, it is important to point out that most of the tissue distribution data were obtained with iodine labeled mAbs and unusually high distribution to thyroid should be interpreted with caution because of the concentration effect of iodine-labeled metabolites in the thyroid.

In cases where an antibody shows high-affinity, high-capacity binding to tissue and target-mediated elimination, the true Vss may be much greater than the distribution volume estimated by standard NCA analysis. It was reported that a mAb against keratin sulfate exhibited much higher tissue concentrations than in the plasma with tissue to blood ratio being 5.9, 4.3, 3.5, and 2.6 for lung, esophagus, kidney, and liver, respectively (Kairemo et al., 2001). Similarly, some mAbs directed against endothelial antigens also showed very high tissue to blood ratios. For example, a tissue distribution study showed that the lung-to-blood ratio was 5, 10, and 15 for antibodies of anti-Thy-1.1, anti-ACE, and anti-ICAM-1, respectively (Danilov et al., 2001). It is worth noting that the non-binding IgG typically has a tissue to blood ratio ranging from 0.04 to 0.68 (Shah & Betts, 2013).

**Figure 6:**
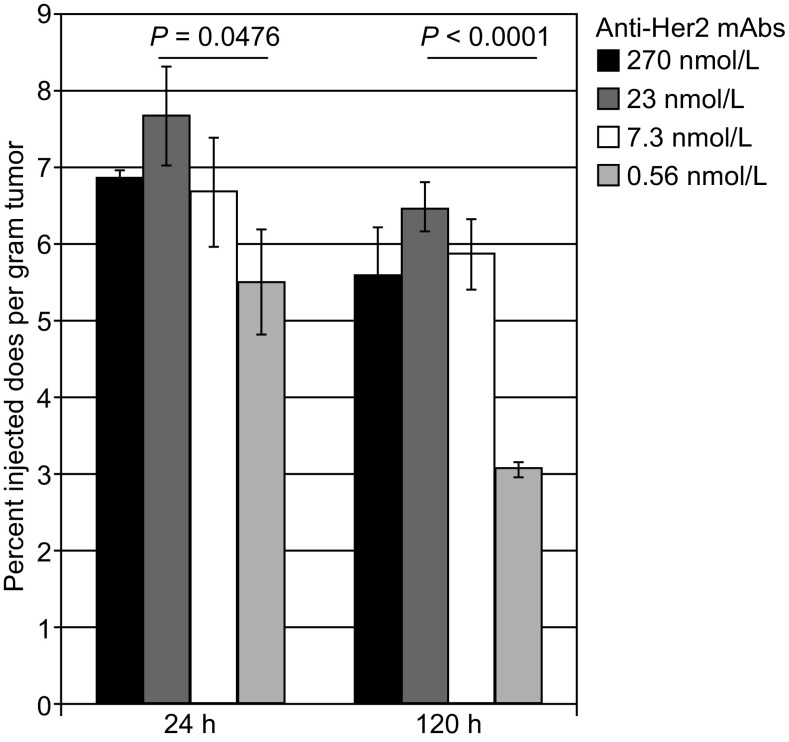
Tumor distribution of anti-Her2 mAbs with different affinities. Adapted from Rudnick et al. (2011)

In a modeling study comparing antibodies with different affinities in distribution to tumors, Fujimori et al. observed that the average antibody concentration in the tumor does not increase linearly with affinity. High antibody affinity at a given dose tends to decrease antibody percolation or penetration to the tumor because the high affinity binding to the edge of the tumor leads to fewer free antibody molecules. This phenomenon was termed “binding site barrier” (Fujimori et al., 1990). Using mAbs that recognize the same Her2 epitope but with different affinities (270, 23, 7.3, and 0.56 nmol/L), Rudnick et al. confirmed the “binding site barrier” phenomenon *in vivo*. They reported that moderate affinity was associated with the highest tumor accumulation at 24 h and 120 h after intravenous injection. High affinity was found to produce low tumor accumulation (Rudnick et al., 2011) (Fig. 6). Similar results were obtained recently in a tumor distribution study by Glatt et al. with anti-CD44 antibodies of different affinities (Glatt et al., 2016).

Zahnd et al. used Darpins, a 14.5 kDa target binding protein to systemically evaluate the size and affinity effects on the tumor distribution. Darpins of different size (with or without Pegylation) and affinity against HER2 were tested in tumor tissue distribution studies. The results showed that for small proteins like Darpin that were eliminated rapidly by kidney filtration, improving affinity directly improved tumor uptake. However, for large targeting agents like Pegylated Darpin, the impact of affinity on tumor accumulation is diminished. The dependence of tumor uptake on binding affinity was found to be weak once *K*d < 100 nmol/L (Wittrup et al., 2012; Zahnd et al., 2010). scFvs, Fabs, diabodies, darpins, and the like are considered too large for sufficiently rapid extravasation and too small to escape renal clearance. Whole IgG antibody is considered to be the optimal size for tumor targeting (Wittrup et al., 2012).

mAb accessibility to tissues can also be observed in PD readouts. In a monkey distribution study with rituximab, the levels of peripheral blood B cells were already depleted by >94% 2 days after the first dose and 9 days after the second dose. However, B cell levels in lymph nodes were only decreased by 42%–57% at 9 days following the 2nd dose (Mao et al., 2013). These results indicate that solid tissues are more difficult to penetrate by mAb and the “binding site barrier” could restrict the distribution of mAb, leading to a delayed pharmacological effect in the tissues.

## **HUMAN PK PREDICTION AND FIRST IN HUMAN STARTING DOSE SELECTION**

PK parameters of small molecule drugs can be scaled across species using a power model of the form Y = a*BW^b^ with reasonable accuracy (Huang & Riviere, 2014; Wang et al., 2016). This equation is based on the principle of allometry; Y is the parameter of interest (e.g., clearance or volume); a is the allometric coefficient; BW is the body weight; b is the allometric exponent. For large molecules such as mAbs with nonlinear PK, assumptions underlying allometric scaling, such as the absence of nonlinear pharmacokinetics and species-specific clearance may not be correct. Nevertheless, in many cases, PK parameters of mAbs with non-target related elimination pathways or doses above the target saturation levels in humans can be reasonably predicted using data from cynomolgus monkeys with an allometric exponent of ~0.85 (Deng et al., 2011; Dong et al., 2011; Ling et al., 2009; Wang & Prueksaritanont, 2010). Different exponents were proposed for reasonable predictions of human CL and Vss for 24 mAbs targeting either soluble antigen or membrane receptors (Oitate et al., 2011). By analyzing data from preclinical and clinical studies of 13 therapeutic mAbs, Deng et al. showed that CL of mAbs can be better predicted based on cynomolgus PK data and an allometric scaling exponent of 0.85. Human concentration–time profiles were also reasonably predicted from the cynomolgus monkey data using species-invariant time method with a fixed exponent of 0.85 for CL and 1.0 for volume of distribution (Deng et al., 2011). A relatively higher accuracy prediction for pharmacokinetics in humans may be achieved by physiologically based PBPK modeling that has the advantage of allowing prediction of antibody levels in many tissues, including tumors. It also takes into consideration the effects of saturable processes (e.g., target binding, FcRn recycling) on antibody PK and the impacts of a variety of other factors (e.g., antigen expression, antibody affinity) on the tissue selectivity of antibody disposition (Shah & Betts, 2013; Wang et al., 2016). However, PBPK models are complex, mathematically difficult to construct, poorly suited to population analyses, and often limited because of a lack of tissue concentration data, parameter availability, or parameter identifiability (Shah & Betts, 2013; Wang et al., 2016). Although the drug exposure in humans can be reasonably predicted for mAbs with linear PK above saturation dose, prediction of drug exposure at low starting doses for mAbs (typically starting dose for dose escalation study) is challenging and generally poor because of nonlinear PK (Dong et al., 2011). Further exploration of species differences in the target expression level, target-antibody binding and target kinetics, and *in vivo* PK studies with relevant dose ranges may help to solve this issue.

**Figure 7:**
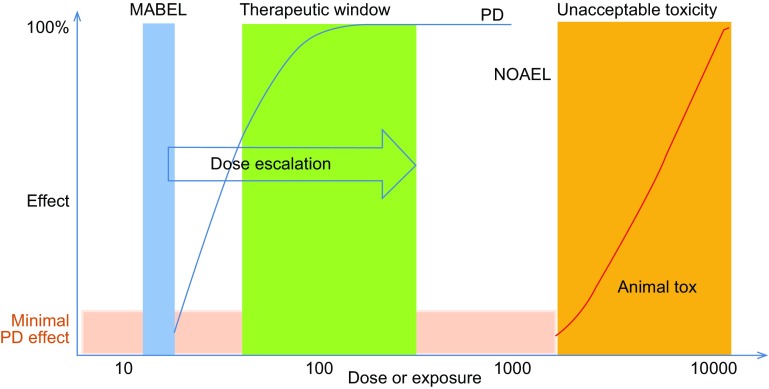
Relationship between MABEL, NOAEL, therapeutic window and toxicity in FIH trial. MABEL: minimally anticipated biological effect level; NOAEL: no observed adverse effect level. Modified from Muller et al., Curr Opin Biotechnol. (2009)

For oncology indications, rule based standard 3 × 3 dose escalation methods have been used for most mAb FIH studies. This scheme is to ensure safety and tolerability in FIH trials. Several approaches coexisted that are frequently used in FIH dose predictions. The classic approach for determining the maximum recommended starting dose (MRSD) is based on the no observed adverse effect level (NOAEL) dose determination from toxicity studies and subsequent human equivalent dose (HED) estimation according to the FDA Guidance (2005). Minimally anticipated biological effect level (MABEL) is EMA’s response to the TGN-1412 disaster which was not predicted with the classical method (EMEA, 2007; Muller et al., 2009; UK Expert Scientific Group, 2006). This approach had been specifically designed for ‘high-risk medicinal products’ with, for example, novel mechanisms of action or high species specificity. Figure 7 graphically depicts the relationship between MABEL, NOAEL, and starting dose. Currently, most clinical development programs use combinations of several or all available approaches to seek ‘totality of evidence’ to develop a FIH dose selection rationale for a particular mAb-based therapeutics. The totality of evidence may include the exposure–response relationships, receptor-occupancy in relationship to efficacy and biological effects and HED based on NOAEL. The MOA should be taken into consideration as well. For example, agonistic or modulator of immune system (high risk) mAbs starting dose should be considered differently from antagonist antibodies. Extreme care should be given to immune agonist antibodies whereas antagonists can generally be tolerated relatively higher starting dose (Luu et al., 2013; Muller et al., 2009). For mAbs that are not considered high-risk biologics, usually antagonist (blocking) mAbs, the MABEL approach may not be required. However, it may still be useful to rationally select the starting human dose from a pharmacological standpoint relative to a toxicological standpoint (Luu et al., 2013). Recently, an FDA oncology analysis of immune activating products and first-in-human dose selection by Saber et al. reinforced the concepts discussed above on the totality of data for considering FIH doses of immune activating biologics (Saber et al., 2016). Importantly, the FDA authors pointed out that while evaluating safe dose is the primary goal of FIH trial, sub-therapeutic doses are not medically justifiable in patients with cancer; therefore, optimization of the FIH trial designs to allow rapid attainment of active therapeutic doses is also important (Saber et al., 2016).

Traditionally, doses of therapeutic mAbs are generally chosen based on body weight. Recently, fixed dosing of mAb is gaining popularity because of dosing convenience in medical practice. The specificity of mAbs, a relatively large therapeutic window and generally a small contribution of body size to pharmacokinetic variability favor fixed dosing of mAbs (Bai et al., 2012; Wang et al., 2009). Using modeling and simulation to compare the PK variations of body weight based dosing vs. fixed dosing, Bai et al. demonstrated that, for most mAbs, body weight had little or moderate effect on PK. The difference of variability in exposure between body weight-based and fixed dosing was generally less than 20%, which is moderate relative to the variability generally observed in pharmacodynamics, efficacy, and safety (Bai et al., 2012). Given the many practical advantages, fixed dosing is generally recommended for FIH studies with mAbs. The dosing strategy in later stages of clinical development could then be determined based on combined knowledge of the body weight effect on pharmacokinetics, safety, and efficacy observed in the early clinical trials (Bai et al., 2012; Wang et al., 2009). For those mAbs that exhibit TMDD and nonlinear pharmacokinetics, loading or ‘induction’ dose strategies may be appropriate to saturate or clear available antigen targets. For example, Herceptin (trastuzumab) has a loading dose of 4 mg/kg loading dose followed by 2 mg/kg weekly dose (Herceptin PI).

## **CONCLUSIONS**

This review article discusses current understanding of PK of therapeutic mAb and Fc-fusion proteins. As a large and polar molecule, mAb and Fc-fusion molecules have PKs that are substantially different from that of small molecule drugs. FcRn mediated recycling is the primary determinant of an IgG antibody’s PK properties. Through Fab and/or Fc engineering, IgG-FcRn interactions can be used to generate a variety of therapeutic antibodies with significantly enhanced half-life or the ability to remove unwanted antigen from circulation (sweeping antibody). Glycosylation on mAb or Fc-fusion protein can have a significant impact on the PK of these molecules. High mannose content is a liability for mAb and sialic acids are beneficial to Fc-fusion proteins. mAb charge can also be important. Variation of pI values by 1–2 units is likely to impact PK, with lower pI values being favorable. In contrast to small molecule drugs, most mAbs display TMDD. This can have significant consequences on study design (both pre-clinical and clinical), in particular on dose selection, dosing scheme, and sampling times. The PK of mAb can also be influenced by anti-drug antibody (ADA) response and off-target binding, which require careful consideration during the discovery stage. mAb is primarily absorbed through the lymphatic system and can be conveniently administered by sc dosing. Large doses or volumes can be administered with hyaluronidase co-formulation. mAb slowly distributes to the interstitial space of tissues via convection. This results in a tissue to blood ratio ranging from 0.1–0.5, although the value can be significantly higher than 1 with mAb showing high-affinity and high-capacity binding in tissues. PK parameters of mAb with linear PK (above the target saturation dose) in humans can be reasonably predicted by using data from cynomolgus monkeys and an allometric exponent of ~0.85. Combination of several methods such as NOAEL and MABEL should be considered for prediction of FIH starting dose. In some situation, fixed dose is possible in humans if body size and weight do not contribute significantly to PK variability.
